# Partial arterial carbon dioxide and oxygen pressure in patients with cardiogenic shock

**DOI:** 10.1007/s11739-025-03926-2

**Published:** 2025-05-09

**Authors:** Jonas Rusnak, Tobias Schupp, Kathrin Weidner, Marinela Ruka, Sascha Egner-Walter, Alexander Schmitt, Muharrem Akin, Kambis Mashayekhi, Mohamed Ayoub, Michael Behnes, Ibrahim Akin

**Affiliations:** 1 Department of Cardiology, Angiology, Haemostaseology and Medical Intensive Care, University Medical Centre Mannheim, Medical Faculty Mannheim, Heidelberg University, Mannheim, Germany; 2 Department of Cardiology, St. Josef-Hospital Ruhr-Universität Bochum, Bochum, Germany; 3 Department of Internal Medicine and Cardiology, Mediclin Heart Centre Lahr, Lahr, Germany; 4 Division of Cardiology and Angiology, Heart Center University of Bochum-Bad Oeynhausen, Bad Oeynhausen, Germany; 5 First Department of Medicine, University Medical Center Mannheim (UMM), Theodor-Kutzer-Ufer 1-3, 68167 Mannheim, Germany

**Keywords:** Cardiogenic shock, Ventilation, Carbon dioxide, Oxygen, Mortality

## Abstract

In patients with acute cardiovascular diseases, hypocapnia, hypoxia and hyperoxia are known to be associated with increased mortality. This monocentric prospective registry study included 238 consecutive patients with cardiogenic shock (CS). The study aimed to assess the prognostic impact of partial arterial carbon dioxide (PaCO_2_) and oxygen pressure (PaO_2_) on 30-day all-cause mortality. Statistical analyses included t-tests, Spearman´s correlation, Kaplan–Meier and Cox regression analyses. No difference was found between quartiles of PaCO_2_ (log-rank p = 0.416) and PaO_2_ (log-rank p = 0.946) in the entire cohort. In the subgroup of patients with ventilation on admission, patients with PaCO_2_ ≤ 33 mmHg showed the highest 30-day all-cause mortality compared to the other quartiles (82.6% vs. 46.9% vs. 54.0% vs. 59.6% log-rank p = 0.026). No differences were found between levels of PaO_2_, when stratified by quartiles (log-rank p = 0.895). After differentiation between patients with PaCO_2_ ≤ 33 mmHg and PaCO_2_ > 33 mmHg the association with 30-day all-cause mortality remained significant (82.6% vs. 54.5% log-rank p = 0.006) in ventilated patients, whereas still no difference could be seen in the entire cohort (log-rank p = 0.264). Even after multivariable adjustment PaCO_2_ ≤ 33 mmHg remained an independent risk factor for 30-day all-cause mortality (HR 1.936; 95% CI 1.131–3.316; p = 0.016) in ventilated CS-patients. In conclusion, no association was found between different levels of PaCO_2_ and PaO_2_ with 30-day all-cause mortality in patients with CS. However, in the subgroup of CS-patients requiring ventilation, PaCO_2_ ≤ 33 mmHg was associated with an increased 30-day all-cause mortality.

**Supplementary Information:**

The online version contains supplementary material available at 10.1007/s11739-025-03926-2.

## Introduction

In patients with cardiogenic shock (CS), respiratory failure and need for ventilation is common and increases mortality [1, 2]. When initiating mechanical ventilation different parameters need to be monitored with assigned target ranges to manage sufficient oxygenation and decarboxylation while maintaining lung protective ventilation. In patients with acute respiratory distress syndrome (ARDS) hypercapnia and respiratory acidosis is common and the consequence of lung protective ventilation [3]. Furthermore, hypocapnia is known to increase risk for short- and long-term mortality as well as worse neurological outcome in patients after cardiac arrest [4–6].

There is little information available about how partial arterial carbon dioxide pressure (PaCO_2_) affects CS patients' outcomes. Several studies investigated the influence of PaCO_2_ on the prognosis in the setting of acute heart failure and it could be demonstrated that patients with hypocapnia have a higher in-hospital and long-term mortality [7–9]. Furthermore, hypocapnia can induce local epicardial coronary artery spasm resulting in T-wave and ST-segment alterations [10, 11].

Partial arterial oxygen pressure (PaO_2_) on the other hand is known to be a crucial factor as hypoxemia and hyperoxemia are associated with an increased mortality in patients with various acute cardiovascular diseases including CS [12–14].

As studies regarding the prognostic influence of PaO_2_ and PaCO_2_ in patients with CS of various causes are limited, the study investigates the influence of PaCO_2_ and PaO_2_ on the 30-day all-cause mortality in this cohort.

## Material and methods

### Study design, inclusion and exclusion criteria

This study derives from an analysis of the “Cardiogenic Shock Registry Mannheim” (CARESMA-registry). The CARESMA-registry represents a prospective single-center registry including consecutive CS-patients who were admitted to the ICU for internal medicine of the University Medical Center Mannheim (UMM), Germany, from June 2019 to May 2021 (clinicaltrials.gov identifier: NCT05575856), as recently published [15]. Patients were eligible for inclusion when CS was the cause of admission for ICU. In detail, this included patients in the emergency department, outpatient clinic, and general ward, as well as patients in the diagnostic area, such as coronary angiography and computed tomography. Besides these, patients with onset of CS outside the hospital, were included. Furthermore, for this analysis solely patients with CS and documented PaCO_2_ and PaO_2_ on admission were included. In 35 patients no documentation of PaCO_2_ and PaO_2_ was available on admission. Therefore, these patients were excluded. No further exclusion criteria were applied. An overview of the inclusion process is illustrated in Supplemental Fig. 1.

**Fig. 1:**
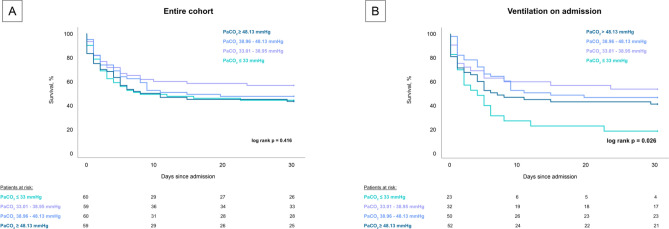
Prognostic impact of PaCO_2_ startified by quartiles on 30-day all-cause mortality in the entire cohort (panel **A**) and the subgroup of ventilated patients (panel **B**)

### Study endpoints

The primary endpoint was 30-day all-cause mortality. All-cause mortality was recorded using the electronic hospital information system and by directly contacting state resident registration offices (‘bureau of mortality statistics’). Identification of patients was verified by name, surname, day of birth, and registered living address. No patient was lost to follow-up with regard to all-cause mortality at 30 days.

The secondary endpoint of the study comprised the correlation of PaCO_2_ on admission with clinically relevant and laboratory parameters.

### Definition of CS

Diagnosis of CS was determined according to the recommendations of the Acute Cardiovascular Care Association of the European Society of Cardiology [16]. Accordingly, CS is defined by hypotension (systolic blood pressure < 90 mmHg) for more than 30 min despite adequate filling status or need for vasopressor or inotropic therapy to achieve systolic blood pressure > 90 mmHg. Additionally, signs for end-organ hypoperfusion must be present such as oliguria with urine output < 30 mL/hour, altered mental status, cold clammy skin, and increased lactate > 2 mmol/L. Moreover, patterns (increased left ventricular end diastolic pressure > 20 mmHg, elevated pulmonary capillary wedge pressure diagnosed by pulmonary artery catheterization or by mitral E-wave deceleration time ≤ 130 ms in echocardiography) or indirect signs (pulmonary congestion confirmed by clinical examination or chest X-ray) of elevated left ventricular filling pressures were mandatory for CS diagnosis. To finally confirm that CS was the fundamental cause of critical illness, a distinct cause of CS was required. This was not limited to acute myocardial infarction. Most recommendations are derived from studies of patients with infarct-related CS. However, data from these studies cannot be transferred to non-infarct-related CS [17, 18]. Therefore, this study included patients with various causes of CS such as arrhythmia, acute decompensated heat failure, pulmonary embolism, valvular heart disease, Tako-Tsubo-cardiomyopathy, pericardial tamponade, and aortic dissection. Cardiac tamponade and pulmonary embolism were classified as CS despite the obstructive mechanism of shock since existing recommendations and guidelines on acute heart failure and CS classify these entities as potential underlying causes for CS [19–22]. Aortic dissection was defined as CS when aortic valve regurgitation was the leading cause for hemodynamic instability [20]. Arrhythmic causes comprised supraventricular arrhythmias such as atrial fibrillation or flutter, atrial tachycardia as well as ventricular tachycardia including primarily ventricular fibrillation.

### Data collection

All relevant clinical data related to the index event were documented using the electronic hospital information system, organizing patient data, including admission documents, vital signs, laboratory values, treatment data and consult notes.

The presence of CS, as well as important laboratory data, ICU-related scores, hemodynamic measurements, ventilator parameters were assessed on the day of admission. Further data being documented contained baseline characteristics, prior medical history, length of index hospital stay, data derived from imaging diagnostics, as well as pharmacological therapies. Documentation of source data was performed by intensivists and ICU nurses during routine clinical care. All procedures were followed in accordance with the ethical standards of the medical ethics committee II of the Medical Faculty Mannheim, University of Heidelberg, Germany and with the Helsinki Declaration of 1964.

Respiratory parameters and intensive care related information were documented by the IntelliSpace Critical Care and anesthesia information system (ICCA, Philips, Philips GmbH Market DACH, Hamburg, Germany) implemented on the ICU and the connected respiratory machines. Furthermore, blood gas samples including PaO_2_ and PaCO_2_ were determined on admission for every patient. Respiratory settings were adjusted by the responsible medical team on the ward to achieve a PaO_2_ goal of > 65 mmHg and a PaCO_2_ that would maintain a pH > 7.20.

### Statistical methods

Quantitative data is presented as median and interquartile range (IQR). They were compared using the Student’s t test for normally distributed data or the Mann–Whitney U test for nonparametric data. Deviations from a Gaussian distribution were tested by the Kolmogorov–Smirnov test. Qualitative data are presented as absolute and relative frequencies and were compared using the Chi-square test or the Fisher’s exact test, as appropriate. Kaplan–Meier analyses on 30-day survival according to the quartiles of PaCO_2_ and PaO_2_ were performed in the entire cohort and in the subgroup of ventilated CS-patients, patients with acute myocardial infarction and acute decompensated heart failure. Univariable hazard ratios (HR) were given together with 95% confidence intervals by performing Cox regression. Thereafter, multivariable Cox regression models were developed using the “backwards selection” option and variables with p-values < 0.10 in the univariable analyses. Furthermore, solely clinically relevant variables such as age, body mass index, sex, chronic obstructive pulmonary disease (COPD), bilirubin, creatinine, troponine I, pH value, norepinephrine dose, cardiac arrest, heart rate, SCAI CS stage, cause of CS, Acute Physiology score, SOFA score, peak inspiratory pressure, peak end-expiratory pressure (PEEP), driving pressure, tidal volume, respiratory rate, mechanical power, lung compliance, PaO_2_ ≥ 65 mmHg and PaO_2_/FiO_2_ on admission were eligible for the multivariable Cox regression model.

Results of all statistical tests were considered significant for p < 0.05. SPSS (Version 29, IBM, Armonk, New York) was used for statistics.

## Results

The final study cohort comprised 238 patients. As presented in Supplemental Table 1, baseline characteristics including measured parameters on admission and prior medical history were nearly equally distributed between survivors and non-survivors. Solely heart rate (85 bpm vs. 95 bpm; p = 0.032) and body temperature (36.1 °C vs. 35.8 °C; p = 0.046) differed between the groups.

As outlined in Table 1, non-survivors had more often an acute myocardial infarction (41.1% vs. 51.6%; p = 0.001) and acute decompensated heart failure (21.4% vs. 28.6%; p = 0.001) as underlying cause of CS. CS severity according to the SCAI classification differed with more patient in stage E (42.9% vs. 65.1%; p = 0.001) and less in stage C (44.6% vs. 26.2%; p = 0.001) in the non-survivor group. Furthermore, severely reduced left ventricular function was more common in non-survivors (36.6% vs. 60.3%; p = 0.002). Patients not surviving were more frequently resuscitated with higher rates of out-of-hospital cardiac arrests (32.1% vs. 44.4%; p = 0.002) and intra-hospital cardiac arrests (10.7% vs. 20.6%; p = 0.002). Moreover, non-survivors were more likely to have non-shockable rhythms (13.4 vs. 38.1%; p = 0.001), showed a longer duration until return of spontaneous circulation (12 min vs. 15 min; p = 0.008) and needed higher doses of norepinephrine (0.1 µg/kg/min vs. 0.2 µg/kg/min; p = 0.001). Use of extracorporeal life support (4.5% vs. 15.9%; p = 0.026), renal replacement therapy (17.9% vs. 45.2%; p = 0.001) and invasive ventilation (51.8% vs. 73.2%; p = 0.015) with higher driving pressure on the respirator (13 cmH_2_O vs. 15 cmH_2_O; p = 0.032) were more common in the non-survivor group. Regarding laboratory parameters on admission, levels of lactate (2.5 mmol/l vs. 4.4 mmol/l; p = 0.001), white blood cells (13.4 × 10^6^/ml vs. 16.5 × 10^6^/ml; p = 0.002), D-Dimer (5.6 mg/l vs. 18.0 mg/l; p = 0.001), and troponin I (0.32 µg/l vs. 1.96 µg/l; p = 0.022) were higher in the non-survivor group, whereas pH values were lower (7.31 vs. 7.27; p = 0.005).

**Table 1:** Shock-related data of patients stratified by survival within 30 days

	Survivor (n = 112)	Non-survivor (n = 126)	p value
Etiology of CS, n (%)			
Acute myocardial infarction	46 (41.1)	65 (51.6)	**0.001**
Arrhythymic	21 (18.8)	6 (4.8)	
Acute decompensated heart failure	24 (21.4)	36 (28.6)	
Valvular heart disease	8 (7.1)	4 (3.2)	
Cardiomyopathy	4 (3.6)	3 (2.4)	
Pulmonary embolism	4 (3.6)	11 (8.7)	
Pericardial tamponade	5 (4.5)	0 (0.0)	
Aortic dissection	0 (0.0)	1 (0.8)	
SCAI shock stage, n (%)			
Stage B	5 (4.5)	0 (0.0)	**0.001**
Stage C	50 (44.6)	33 (26.2)	
Stage D	9 (8.0)	11 (8.7)	
Stage E	48 (42.9)	82 (65.1)	
Transthoracic echocardiography			
LVEF > 55%, (n, %)	15 (13.4)	10 (7.9)	**0.002**
LVEF 54–41%, (n, %)	19 (17.0)	11 (8.7)	
LVEF 40–30%, (n, %)	33 (29.5)	24 (19.1)	
LVEF < 30%, (n, %)	41 (36.6)	76 (60.3)	
LVEF not documented, (n, %)	4 (3.6)	5 (4.0)	
Vena cava inverior, cm [median, (IQR)]	1.8 (1.5–2.2)	1.8 (1.6–2.2)	0.494
TAPSE, mm [median, (IQR)]	17 (12–20)	15 (12–17)	0.356
Cardiopulmonary resuscitation			
Out-of-hospital cardiac arrest, n (%)	36 (32.1)	56 (44.4)	**0.002**
In-hospital cardiac arrest, n (%)	12 (10.7)	26 (20.6)	
Shockable rhythm, n (%)	32 (28.6)	34 (27.0)	0.785
Non-shockable rhythm, n (%)	15 (13.4)	48 (38.1)	**0.001**
ROSC, min (median, IQR)	12 (5–20)	15 (10–30)	**0.008**
Respiratory status			
Invasive ventilation on admission	58 (51.8)	88 (73.2)	**0.015**
Non-invasive ventilation on admission	7 (6.3)	4 (3.2)	0.357
Duration of ventilation, days, (median, (IQR)]	2 (0–8)	2 (1–5)	0.145
PaO_2_/FiO_2_ ratio, [median, (IQR)]	238 (147–367)	214 (124–336)	0.308
PaCO_2_, mmHg [median, (IQR)]	45.1 (38.7–51.1)	39.8 (32.2–49.2)	0.912
PEEP, cmH_2_O [median, (IQR)]	7 (5–8)	8 (5–10)	0.644
Driving pressure, cmH_2_O [median, (IQR)]	13 (10–17)	15 (12–17)	**0.032**
Compliance [ml/cmH_2_O (median, (IQR)]	35.2 (25.8–43.8)	29.2 (23.1–37.6)	0.056
Inspiratory time, sec [median, (IQR)]	1.1 (1.0–1.4)	1.1 (1.0–1.4)	0.530
Multiple organ support during ICU			
Norepinephrine dose on admission, µg/kg/min [median, (IQR)]	0.1 (0.1–0.2)	0.2 (0.1–0.7)	**0.001**
Dobutamin, cumulative dose day 1, mg/kg	11 (4–20)	4 (2–13)	0.145
Extracorporeal life support, n (%)	5 (4.5)	20 (15.9)	**0.026**
Renal replacement therapy, n (%)	20 (17.9)	57 (45.2)	**0.001**
Baseline laboratory values, [median, (IQR)]			
pH	7.31 (7.23–7.37)	7.27 (7.16–7.36)	**0.005**
Lactate (mmol/l)	2.5 (1.6–3.8)	4.4 (2.3–10.2)	**0.001**
Sodium (mmol/l)	138 (136–140)	138 (136–142)	0.415
Potassium (mmol/l)	4.2 (3.7–4.8)	4.4 (3.9–5.1)	0.355
Creatinine (mg/dl)	1.3 (1.1–1.8)	1.6 (1.2–2.3)	0.056
Hemoglobin (g/dl)	12.3 (10.1–14.2)	12.4 (10.3–13.9)	0.714
White blood cells (10^6^/ml)	13.4 (9.9–17.8)	16.5 (12.6–20.2)	**0.002**
Platelets (10^6^/ml)	220 (157–288)	231 (178–267)	0.735
INR	1.1 (1.1–1.3)	1.2 (1.1–1.5)	0.320
d-Dimer (mg/l)	5.6 (2.0–14.3)	18.0 (5.9–32.0)	**0.001**
AST (U/l)	110 (38–218)	185 (64–524)	0.790
ALT (U/l)	53 (30–133)	103 (40–323)	0.323
Bilirubin (mg/dl)	0.61 (0.42-.096)	0.64 (0.46–1.00)	0.462
Troponin I (µg/l)	0.32 (0.09–2.49)	1.96 (0.33–12.44)	**0.022**
NT-pro BNP (pg/ml)	4480 (454–12,343)	6100 (1098–14,262)	0.210
Procalcitonin (ng/ml)	0.31 (0.07–0.67)	0.28 (0.16–1.09)	0.608
C-reactive protein (mg/l)	12.0 (4.0–49.8)	16.0 (4.0–41.5)	0.642

Level of significance p < 0.05. Bold type indicates statistical significance

ALT, alanine aminotransferase; AST, aspartate aminotransferase; CS, cardiogenic shock; FiO_2_, fraction of inspired oxygen; IQR, interquartile range; LVEF, left ventricular ejection fraction; NT-pro BNP, aminoterminal pro-B-type natriuretic peptide; PaO_2_, partial pressure of oxygen; PaCO_2_, partial pressure of carbon dioxide; PEEP, positive end-expiratory pressure; ROSC, return of spontaneous circulation; SCAI, Society for Cardiovascular Angiography and Interventions; TAPSE, tricuspid annular plane systolic excursion

As illustrated in Fig. 1, after differentiation in quartiles of PaCO_2_ (≤ 33 mmHg vs. 33.01–38.95 mmHg vs. 38.96–48.13 mmHg vs. > 48.13 mmHg) the risk of 30-day all-cause mortality was nearly equally distributed (log-rank p = 0.416). However, in the subgroup of CS-patients with ventilation on admission, CS-patients in the lowest quartile (PaCO_2_) showed the highest 30-day all-cause mortality (82.6% vs. 46.9% vs. 54.0% vs. 59.6% log-rank p = 0.026).

**Fig. 2:**
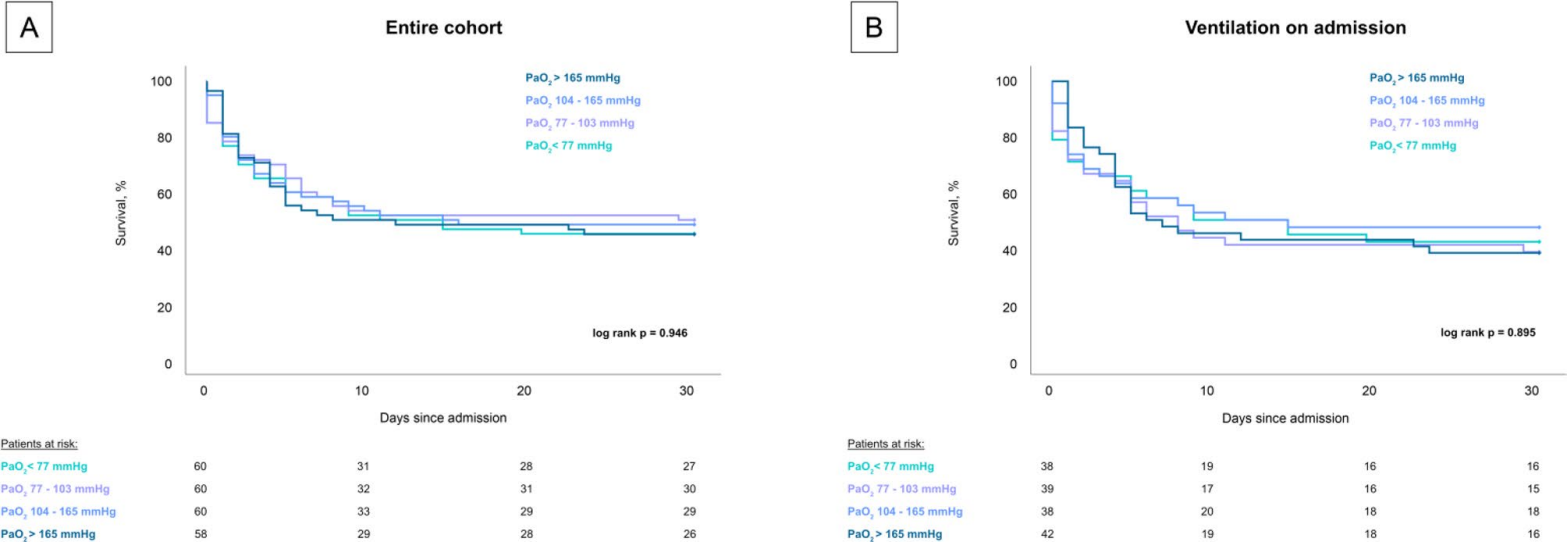
Prognostic impact of PaO_2_ startified by quartiles on 30-day all-cause mortality in the entire cohort (panel **A**) and the subgroup of ventilated patients (panel **B**)

In contrast, after differentiating patients in quartiles of PaO_2_ (< 77 mmHg vs. 77–103 mmHg vs. 104–165 mmHg vs. > 165 mmHg) no association could be seen in the entire cohort (log-rank p = 0.946) and the subgroup of CS-patients with ventilation on admission (log-rank p = 0.895) (Fig. 2).

**Fig. 3:**
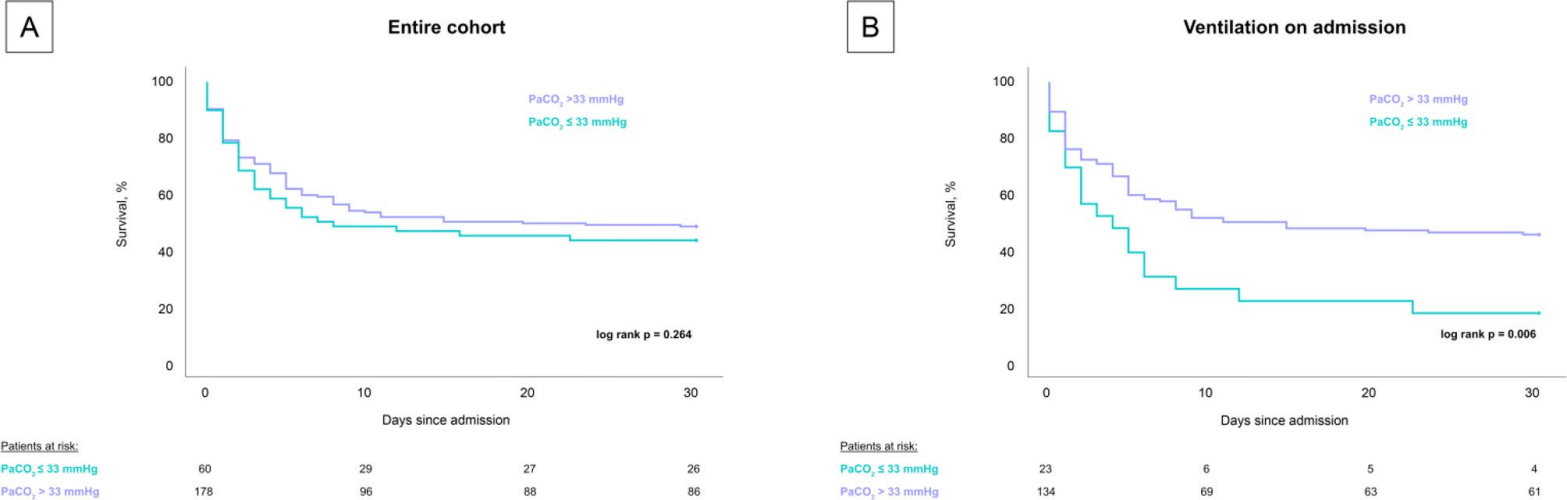
Prognostic impact of PaCO_2_ ≤ 33 mmHg compared to PaCO_2_ > 33 mmHg on 30-day all-cause mortality in the entire cohort (panel **A**) and the subgroup of ventilated patients (panel **B**)

After differentiation between patients with PaCO_2_ ≤ 33 mmHg and PaCO_2_ > 33 mmHg, the group with the lower PaCO_2_ still showed a significantly higher 30-day all-cause mortality in the ventilated group (82.6% vs. 54.5%, log-rank p = 0.006) whereas no significant difference could be seen in the entire cohort (56.7% vs. 51.7%, log-rank p = 0.264) (Fig. 3).

These findings were confirmed in Cox regression analyses, in which PaCO_2_ ≤ 33 mmHg (HR 1.163; 95% CI 0.785–1.724; p = 0.451) showed no significant association in the entire cohort (Table 2), whereas levels of creatinine (HR 1.114; 95% CI 1.012–1.227; p = 0.028), troponine I (HR 1.002; 95% CI 1.001–1.004; p = 0.001), pH values (HR 0.063; 95% CI 0.015–0.261; p = 0.001), doses of norepinephrine on admission (HR 1.367; 95% CI 1.204–1.550; p = 0.001), cardiac arrest on admission (HR 1.809; 95% CI 1.253–2.610; p = 0.002), heart rate (HR 1.008; 95% CI 1.002–1.015; p = 0.011), SCAI CS stage (HR 1.422; 95% CI 1.172–1.726; p = 0.001), Acute Physiology score (HR 1.061; 95% CI 1.034–1.090; p = 0.001) and SOFA score (HR 1.105; 95% CI 1.053–1.160; p = 0.001) were associated with the primary endpoint.

**Table 2:** Univariable Cox regression analyses with regard to 30-day all-cause mortality in the entire cohort

Variables	Univariable		
	HR	95% CI	p value
Age	1.006	0.993–1.019	0.343
Body mass index (kg/m^2^)	1.014	0.982–1.047	0.389
Sex (male)	0.980	0.686–1.400	0.911
COPD	1.112	0.734–1.684	0.617
Bilirubin (mg/dl)	0.995	0.846–1.169	0.947
Creatinine (mg/dl)	1.114	1.012–1.227	**0.028**
Troponine I (µg/l)	1.002	1.001–1.004	**0.001**
pH	0.063	0.015–0.261	**0.001**
Norepinephrine on admission (µg/kg/min)	1.367	1.204–1.550	**0.001**
Cardiac arrest on admission	1.809	1.253–2.610	**0.002**
Heart rate (bpm)	1.008	1.002–1.015	**0.011**
Respiratory rate (breaths/min)	1.025	0.996–1.054	0.088
SCAI CS stage	1.422	1.172–1.726	**0.001**
Cause of CS	0.980	0.876–1.096	0.719
Acute Physiology score	1.061	1.034–1.090	**0.001**
SOFA score	1.105	1.053–1.160	**0.001**
Peak inspiratory pressure (cmH_2_O)	1.030	0.999–1.063	0.055
PEEP (cmH_2_O)	1.038	0.958–1.126	0.362
Driving pressure (cmH_2_O)	1.033	0.997–1.070	0.069
Tidal volume (ml)	0.999	0.998–1.001	0.548
Lung compliance (ml/cmH_2_O)	0.987	0.975–1.000	0.055
Mechanical power (J/min)	1.014	0.991–1.037	0.234
PaO_2_ ≥ 65 mmHg	0.743	0.440–1.256	0.267
PaO_2_/FiO_2_ ratio	1.000	0.999–1.001	0.414
PaCO_2_ ≤ 33 mmHg	1.163	0.785–1.724	0.451

Bpm, beats per minute; CI, confidence interval; COPD, chronic obstructive pulmonary disease; CS, cardiogenic shock; FiO_2_, fraction of inspired oxygen; HR, hazard ratio; PaCO_2_, partial pressure of carbon dioxide; PaO_2_, partial pressure of oxygen; PEEP, positive end expiratory pressure; SCAI, society for Cardiovascular Angiography and Interventions;

Level of significance p < 0.05. Bold type indicates statistical significance

^a^Variables in the multivariable regression model: norepinephrine, SCAI CS stage, troponine I, creatinine, driving pressure, peak inspiratory pressure, lung compliance, Acute Physiology score, PaCO_2_ ≤ 33 mmHg, cardiac arrest on admission;

As outlined in Table 3, regarding solely the subgroup of ventilated patients, PaCO_2_ ≤ 33 mmHg showed an association with higher 30-day all-cause mortality (HR 1.955; 95% CI 1.177–3.247; p = 0.010), which was still significant after multivariable adjustment (HR 1.936; 95% CI 1.131–3.316; p = 0.016). In this multivariable Cox regression model, troponine levels (HR 1.002; 95% CI 1.000–1.003; p = 0.012), norepinephrine doses on admission (HR 1.225; 95% CI 1.030–1.456; p = 0.022) and Acute Physiology score (HR 1.054; 95% CI 1.009–1.102; p = 0.019) were as well associated with the primary endpoint.

**Table 3:** Uni- and multivariable Cox regression analyses with regard to 30-day all-cause mortality in ventilated CS-patients

Variables	Univariable			Multivariable^a^		
	HR	95% CI	p value	HR	95% CI	p value
Age	1.007	0.992–1.021	0.372	–	–	–
Body mass index (kg/m^2^)	1.007	0.968–1.047	0.739	–	–	–
Sex (male)	1.085	0.715–1.646	0.702	–	–	–
COPD	1.023	0.629–1.666	0.926	–	–	–
Bilirubin (mg/dl)	0.898	0.683–1.182	0.444	–	–	–
Creatinine (mg/dl)	1.111	0.980–1.260	0.099	–	–	–
Troponine I (µg/l)	1.002	1.001–1.003	**0.002**	1.002	1.000–1.003	**0.012**
pH	0.105	0.019–0.580	**0.010**	–	–	**–**
Norepinephrine on admission (µg/kg/min)	1.286	1.108–1.492	**0.001**	1.225	1.030–1.456	**0.022**
Cardiac arrest on admission	2.299	1.277–4.139	**0.006**	–	–	–
Heart rate (bpm)	1.001	0.994–1.009	0.722	–	–	–
SCAI CS stage	1.575	1.138–2.180	**0.006**	–	–	–
Cause of CS	1.039	0.914–1.182	0.554	–	–	–
Acute Physiology score	1.070	1.029–1.114	**0.001**	1.054	1.009–1.102	**0.019**
SOFA score	1.056	0.984–1.133	0.129	–	–	–
Peak inspiratory pressure (cmH_2_O)	1.031	1.000–1.064	0.053	–	–	–
PEEP (cmH_2_O)	1.038	0.957–1.127	0.369	–	–	–
Driving pressure (cmH_2_O)	1.034	0.998–1.071	0.066	–	–	–
Tidal volume (ml)	0.999	0.998–1.001	0.522	–	–	–
Lung compliance (ml/cmH_2_O)	0.987	0.974–1.000	0.050	–	–	–
Respiratory rate (breaths/min)	1.013	0.979–1.049	0.467	–	–	–
Mechanical power (J/min)	1.014	0.992–1.037	0.219	–	–	–
PaO_2_ ≥ 65 mmHg	0.807	0.391–1.667	0.563	–	–	–
PaO_2_/FiO_2_ ratio	1.000	0.999–1.001	0.855	–	–	–
PaCO_2_ ≤ 33 mmHg	1.955	1.177–3.247	**0.010**	1.936	1.131–3.316	**0.016**

Bpm, beat per minute; CI, confidence interval; COPD, chronic obstructive pulmonary disease; CS, cardiogenic shock; FiO_2_, fraction of inspired oxygen; HR, hazard ratio; PaCO_2_, partial pressure of carbon dioxide; PaO_2_, partial pressure of oxygen; PEEP, positive end expiratory pressure; SCAI, society for Cardiovascular Angiography and Interventions;

Level of significance p < 0.05. Bold type indicates statistical significance

^a^Variables in the multivariable regression model: pH; norepinephrine, SCAI CS stage, troponine I, creatinine, driving pressure, peak inspiratory pressure, lung compliance, Acute Physiology score, PaCO_2_ ≤ 33 mmHg, cardiac arrest on admission;

Consistent with the findings from the analysis of the entire cohort, no significant differences were observed in the subgroup of patients with acute myocardial infarction or acute decompensated heart failure when stratified by quartiles or median (Supplemental Fig. 2 and Supplemental Fig. 3). After including only patients on mechanical ventilation, those with acute myocardial infarction and a PaCO_2_ ≤ 33 mmHg had significantly higher 30-day all-cause mortality compared to those with higher PaCO_2_ levels (log-rank 0.045) (Supplemental Fig. 4). However, among ventilated patients with acute decompensated heart failure, no difference in mortality was observed when stratified by quartiles or median of PaCO_2_ levels (Supplemental Fig. 5).

Supplemental Table 2 outlines the correlation of PaCO_2_ with clinical and laboratory data in ventilated CS-patients. In this analysis PaCO_2_ significantly correlated indirectly with age (r = − 0.190; p = 0.017), pH values (r = − 0.485; p = 0.001), PaO_2_/FiO_2_ ratio (r = − 0.297; p = 0.001) and lung compliance (r = − 0.235; p = 0.005), whereas a direct correlation could be seen for heart rate (r = 0.174; p = 0.029), driving pressure (r = 0.296; p = 0.001) and peak inspiratory pressure (r = 0.305; p = 0.001).

After stratifying patients based on ventilation and PaCO_2_ levels (≤ 33 mmHg vs. > 33 mmHg), the ventilator settings were largely similar between the two groups, except for driving pressure (12 cmH_2_O vs. 15 cmH_2_O; p = 0.032) and mechanical power (15.0 J/min vs. 18.2 J/min; p = 0.040), both of which were higher in the group with PaCO_2_ > 33 mmHg (Supplemental Table 3). Furthermore, no difference could be found in pulmonary comorbidities detected in X-ray or computed tomography.

## Discussion

The present study evaluates the prognostic impact of PaCO_2_ and PaO_2_ in patients with CS. In this cohort PaCO_2_ and PaO_2_ showed no association with 30-day all-cause mortality after stratifying by quartiles. However, in the subgroup of ventilated patients PaCO_2_ ≤ 33 mmHg was n independent risk factor for an increased 30-day all-cause mortality in Kaplan Meier analysis, whereas PaO_2_ showed no differences regarding the 30-day all-cause mortality when stratified by quartiles. These results remained consistent even after multivariable adjustment, which included relevant ventilatory parameters such as driving pressure, peak inspiratory pressure, and lung compliance.

Both hypo- and hyperoxia are known to negatively affect the outcome and mortality of patients with acute cardiovascular disease, such as acute myocardial infarction, acute heart failure, and CS [12–14]. In the present study, no association was observed between PaO_2_ on admission and 30-day all-cause mortality when stratified by quartiles. Hypo- and hypercapnia have been linked to influence prognosis in patients with cardiovascular disease and critical illness by altering the lung’s ventilation/perfusion match, intracranial pressure, myocardial contraction, and vascular resistance [6, 23–25]. In patients with cardiac arrest hypocapnia is associated with an increased mortality and worse neurological outcome, and hypercapnia is suspected to be beneficial by increasing cerebral blood flow [5, 24]. However, in the recently published Targeted Therapeutic Mild Hypercapnia after Resuscitated Cardiac Arrest (TAME) trial of 1668 patients with cardiac arrest, it could be demonstrated that there was no benefit or harm of mild hypercapnia on mortality and neurological outcome after six months [4]. A pre-planned sub-analysis of 84 patients from the TAME trial revealed that patients in the hypercapnia group had a significantly higher cardiac index and higher cardiac power output, primarily due to higher stroke volumes [26]. The authors postulate that these findings might be induced by lower ventilator settings, which could have increased venous return. This shows that mechanical ventilation has major implications in critical-ill cardiac patients.

Alterations of PaCO_2_ are commonly observed in patients with cardiovascular disease, particularly in patients with acute heart failure [27]. Observational studies could demonstrate that low PaCO_2_ is associated with increased in-hospital and one-year mortality in patients with acute heart failure [7, 8]. Specifically, patients with PaCO_2_ < 32.3 mmHg exhibited the highest in-hospital mortality in a retrospective analysis of patients with acute heart failure [7]. These findings align with large retrospective analyses of patients with respiratory failure due to cardiac causes, such as cardiac pulmonary edema and the need for non-invasive ventilation, which also showed higher in-hospital or one-year mortality in the presence of hypocapnia [28, 29]. However, all of these studies excluded patients with CS or those requiring invasive mechanical ventilation, so the results cannot be transferred to the specific cohort of patients with CS.

Therefore, in addition to patients with acute heart failure and absence of CS as well as patients with cardiac arrest, the present study demonstrates that in the cohort of ventilated patients with CS of various causes, a PaCO_2_ ≤ 33 mmHg is independently associated with increased 30-day all-cause mortality. The underlying mechanisms of these results might be multifactorial. By tolerating higher CO_2_ values lower ventilator settings are required, which may increase venous return and consequently raise cardiac output by higher stroke volume [26]. With lower ventilator settings, a lung protective ventilation can be achieved, reducing negative implications of ventilator-induced lung injury [30–33]. However, as CO_2_ levels correlated directly with driving pressure and peak inspiratory pressure, it can be assumed that lower ventilator settings were not the main cause of improvement in patients with lower PaCO_2_. Furthermore, ventilated patients with PaCO_2_ ≤ 33 mmHg were exposed to lower driving pressure and mechanical power, highlighting that lower ventilatory settings and ventilator-induced lung injury were not the primary factor contributing to the worse outcome in patients with PaCO_2_ ≤ 33 mmHg. Therefore, it is more likely that hypocapnia itself is the primary factor contributing to a worse outcome. Hypocapnia can reduce coronary blood flow and increase the risk of coronary vasospasms [23–25, 34]. This may worsen the outcome of patients with acute coronary syndrome and acute myocardial infarction by decreasing myocardial oxygen supply and worsening ischemic injuries [24, 35]. This is reflected by the subgroup analyses of ventilated patients with acute myocardial infarction, in which it could be demonstrated that PaCO_2_ ≤ 33 mmHg was associated with increased 30-day all-cause mortality. Furthermore, hypocapnia is known to increase systemic vascular resistance, which could lead to an increase in myocardial afterload [24]. Finally, hypercapnic acidosis has been shown to suppress immune responses and may reduce inflammation [3]. Therefore, normal or hypercapnic PaCO_2_ levels may help mitigate the ongoing inflammation process in patients with CS, whereas hypocapnia could exacerbate inflammation and worsen the CS shock spiral.

As patients with CS primarily face a cardiocirculatory issue, less attention is typically given to ventilatory strategies in daily clinical practice. However, since hypocapnia can have negative cardiovascular effects, greater attention should be paid to PaCO_2_ levels in this patient cohort. By allowing higher PaCO_2_ levels, the harmful effects of hypocapnia can be minimized. Therefore, PaCO_2_ levels should be kept above 33 mmHg, and the invasiveness of ventilation should be reduced to the lowest possible level to avoid potentially harmful hypocapnia. This study has several limitations. Due to the single-center and observational design, the results may be influenced by both measured and unmeasured confounders, although we adjusted for potential confounders using multivariate Cox regression. These findings should be considered as hypothesis-generating, as a causal correlation cannot be demonstrated due to the observational study design. Hypocapnia with PaCO_2_ ≤ 33 mmHg may therefore be a useful risk predictor in CS, but it should always be considered as a supplementary parameter alongside other risk factors and scores, and never used as an isolated predictor. The subgroup of ventilated patients was small, so the results should be interpreted with caution due to the limited sample size. This is particularly evident in the subgroup analysis of ventilated patients with acute decompensated heart failure as the underlying cause of CS. Furthermore, echocardiographic and invasive measurements, particularly those assessing right and left ventricular function and peripheral vascular resistance, were inconsistent and could not be included in the analysis.

In conclusion, the present study could demonstrate that in patients with CS no association was found between different levels of PaCO_2_ and PaO_2_ with 30-day all-cause mortality. However, ventilated patients with CS and PaCO_2_ ≤ 33 mmHg revealed a significantly higher 30-day all-cause mortality. Even after multivariable adjustment PaCO_2_ ≤ 33 mmHg remained an independent risk predictor in patients with CS and mechanical ventilation. These findings give new insights on potential impacts of PaCO_2_ in the specific cohort of patients with CS and call for more thorough research in larger clinical trials.

## Supplementary Information

Below is the link to the electronic supplementary material.

Supplementary file1 (DOCX 596 KB)

## Data Availability

The data that support the findings of this study are available from the corresponding author upon reasonable request.
