# Supplementary material for: Partial arterial carbon dioxide and oxygen pressure in patients with cardiogenic shock
Source: Intern Emerg Med. 2025 May 9;20(4):1077–86. doi: 10.1007/s11739-025-03926-2 (PMC12130108; doi:10.1007/s11739-025-03926-2)

| **Supplemental Table 1. Baseline characteristics stratified by survival within 30 days** | | | | | |
| --- | --- | --- | --- | --- | --- |
|  | **Survivor**  (n = 112) | | **Non-survivor**  (n = 126) | | **p value** |
| **Age** (median, (IQR)) | 72 | (62-79) | 74 | (64-81) | 0.354 |
| **Male sex,** n (%) | 66 | (58.9) | 76 | (60.3) | 0.827 |
| **Body mass index,** kg/m^2^ (median, (IQR)) | 26.0 | (23.9-28.0) | 26.6 | (24.5-30.5) | 0.132 |
| **Clinical parameters,** (median, (IQR)) |  |  |  |  |  |
| Body temperature (°C) | 36.1 | (35.2-36.6) | 35.8 | (34.5-36.5) | **0.046** |
| Heart rate (bpm) | 85 | (69-106) | 95 | (74-112) | **0.032** |
| Systolic blood pressure (mmHg) | 110 | (94-130) | 105 | (93-127) | 0.281 |
| Respiratory rate (breaths/min) | 20 | (16-22) | 20 | (17-25) | 0.180 |
| **Cardiovascular risk factors,** *n* (%) |  |  |  |  |  |
| Arterial hypertension | 82 | (73.2) | 90 | (71.4) | 0.759 |
| Diabetes mellitus | 38 | (33.9) | 50 | (39.7) | 0.359 |
| Hyperlipidemia | 58 | (51.8) | 64 | (50.8) | 0.879 |
| Smoking | 41 | (36.6) | 46 | (36.5) | 0.987 |
| **Prior medical history,** *n* (%) |  |  |  |  |  |
| Coronary artery disease: | 39 | (34.8) | 46 | (36.5) | 0.786 |
| 1-vessel disease | 9 | (8.0) | 14 | (11.1) | 0.560 |
| 2-vessel disease | 6 | (5.4) | 3 | (2.4) |  |
| 3-vessel disease | 24 | (21.4) | 29 | (23.0) |  |
| Congestive heart failure | 39 | (34.8) | 47 | (37.3) | 0.691 |
| Atrial fibrillation | 35 | (31.3) | 40 | (31.7) | 0.934 |
| Chronic kidney disease | 39 | (34.8) | 43 | (34.1) | 0.910 |
| Stroke | 18 | (16.1) | 15 | (11.9) | 0.353 |
| COPD | 19 | (17.0) | 29 | (23.0) | 0.246 |
| Liver cirrhosis | 6 | (5.4) | 2 | (1.6) | 0.152 |
| **Medication on admission**, *n* (%) |  |  |  |  |  |
| ACE-inhibitor | 40 | (35.7) | 39 | (31.0) | 0.436 |
| ARB | 20 | (17.9) | 22 | (17.5) | 0.936 |
| Beta-blocker | 58 | (51.8) | 61 | (48.5) | 0.603 |
| ARNI | 5 | (4.5) | 3 | (2.4) | 0.482 |
| Mineralocorticoid antagonist | 18 | (16.1) | 18 | (14.3) | 0.701 |
| SGLT-2 inhibitor | 4 | (3.6) | 4 | (3.2) | 1.000 |
| Diuretics | 47 | (42.0) | 58 | (46.0) | 0.528 |
| ASA | 31 | (27.7) | 35 | (27.8) | 0.986 |
| P2Y12-inhibitor | 10 | (8.9) | 12 | (9.5) | 0.874 |
| Statin | 55 | (49.1) | 49 | (38.9) | 0.113 |
| \| ACE, angiotensin-converting-enzyme; ARB, angiotensin receptor blocker; ARNI, angiotensin receptor neprilysin inhibitor; ASA, acetylsalicylic acid; bpm, beats per  minute; COPD, chronic obstructive pulmonary disease; DP, driving pressure; IQR, interquartile range; Level of significance p<0.05. Bold type indicates statistical significance. \| \| --- \| | | | | | |

| **Supplemental Table 2** Correlations of PaCO_2_ with laboratory and clinical parameters in ventilated CS-patients. | | |
| --- | --- | --- |
|  | PaCO_2_ | |
|  | r | p value |
| Age | -0.190 | 0.017 |
| Body mass index (kg/m^2^) | 0.054 | 0.502 |
| Heart rate (bpm) | 0.174 | 0.029 |
| Lactate (mmol/L) | -0.051 | 0.525 |
| Norepinephrine dose (µg/kg/min) | -0.064 | 0.428 |
| pH | -0.485 | 0.001 |
| PaO_2_/FiO_2_ ratio | -0.297 | 0.001 |
| PEEP (cmH_2_O) | 0.107 | 0.198 |
| Driving pressure (cmH_2_O) | 0.296 | 0.001 |
| Peak inspiratory pressure (cmH_2_O) | 0.305 | 0.001 |
| Tidal volume (ml) | -0.097 | 0.245 |
| Respiratory rate (breaths/min) | 0.039 | 0.634 |
| Lung compliance (ml/cmH_2_O) | -0.235 | 0.005 |
| Pneumonia (ICU acquired) | 0.047 | 0.555 |
| Bpm, beats per minute; FiO2, fraction of inspired oxygen; ICU, intensive care unit; PaO2, partial pressure of oxygen; PaCO2, partial pressure of carbon dioxide; PEEP, positive end expiratory pressure;  Level of significance p<0.05. Bold type indicates statistical significance. | | |

**Supplemental Figure 1** In- and exclusion process


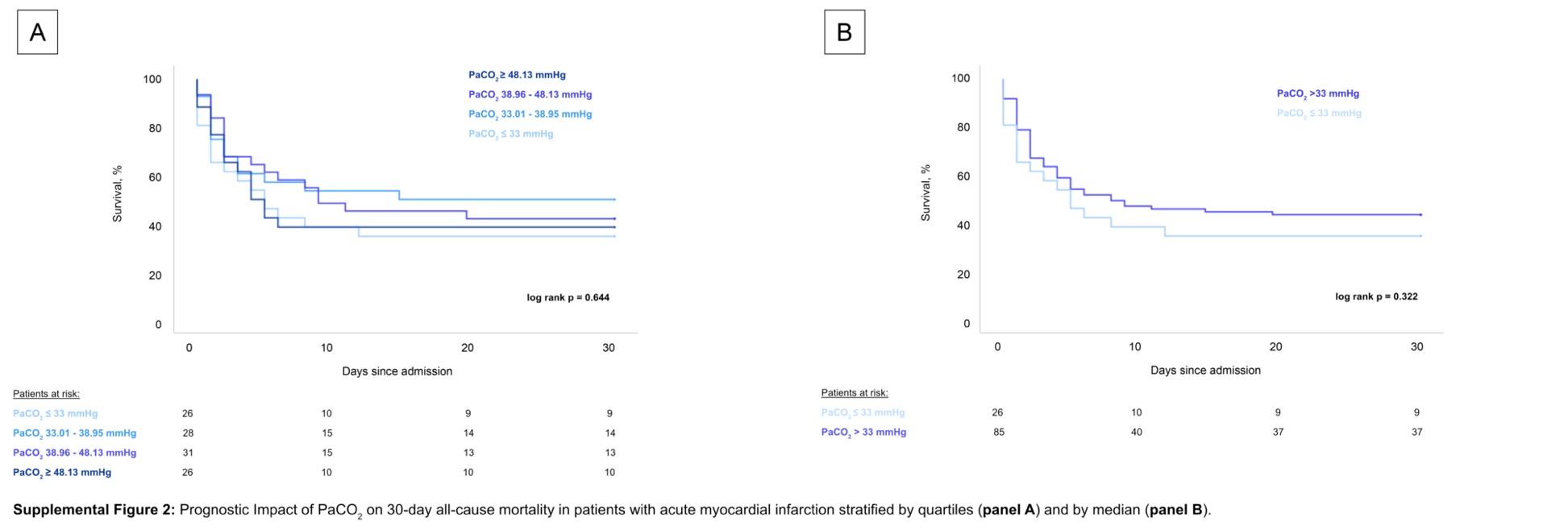


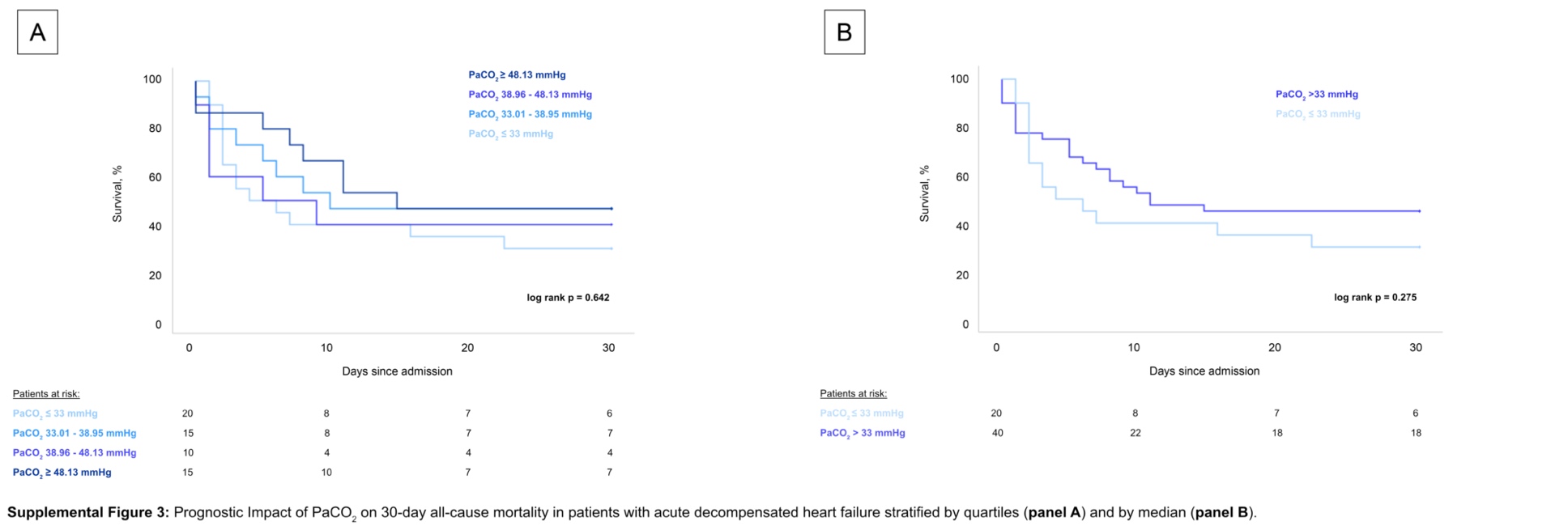


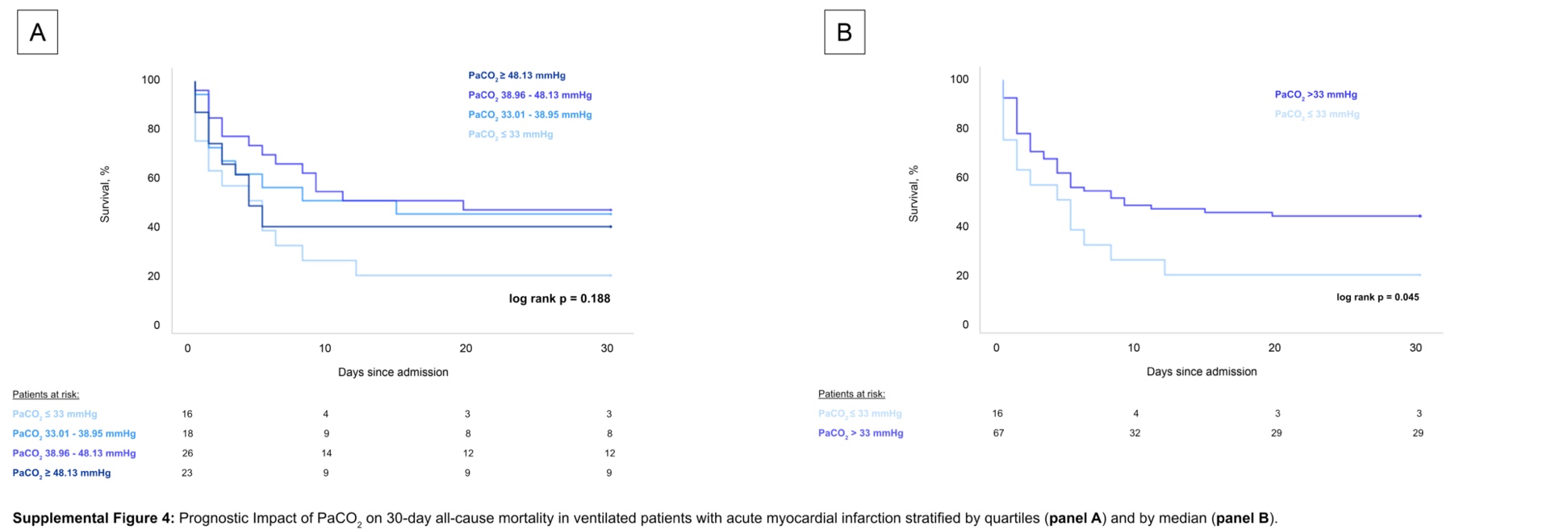


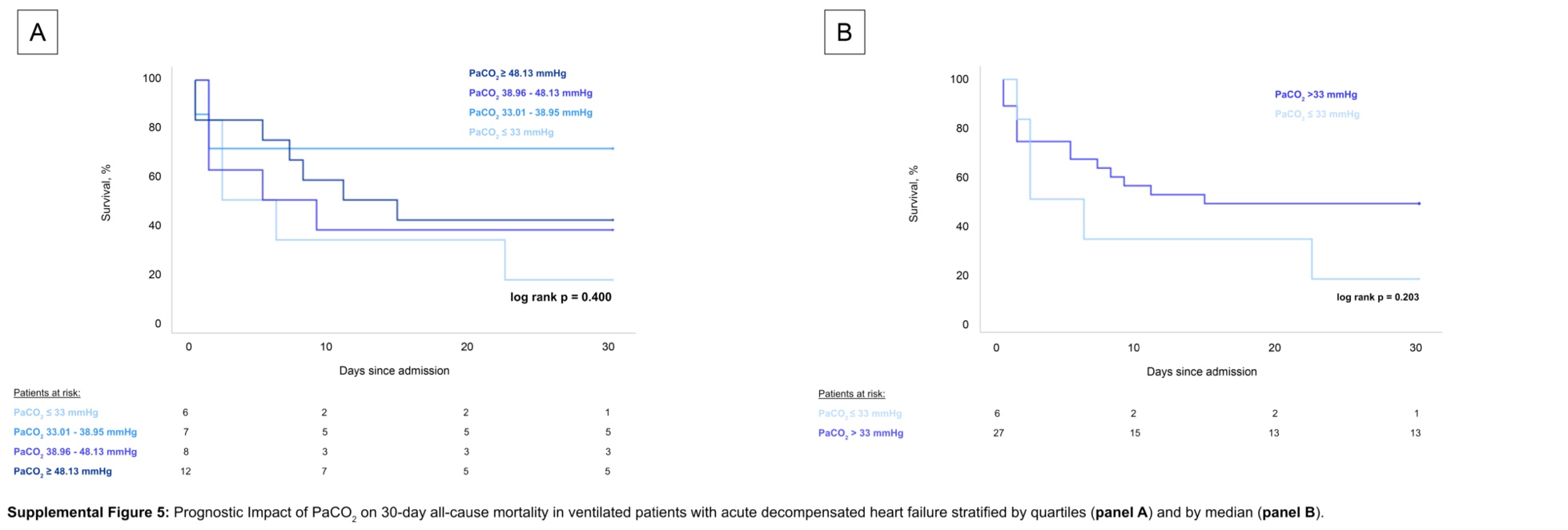

Supplement: Supplementary file 1 — Supplementary file1 (DOCX 596 KB) [file 11739_2025_3926_MOESM1_ESM.docx]
